# Phenoloxidase Activity Acts as a Mosquito Innate Immune Response against Infection with Semliki Forest Virus

**DOI:** 10.1371/journal.ppat.1002977

**Published:** 2012-11-08

**Authors:** Julio Rodriguez-Andres, Seema Rani, Margus Varjak, Margo E. Chase-Topping, Markus H. Beck, Mhairi C. Ferguson, Esther Schnettler, Rennos Fragkoudis, Gerald Barry, Andres Merits, John K. Fazakerley, Michael R. Strand, Alain Kohl

**Affiliations:** 1 The Roslin Institute and Royal (Dick) School of Veterinary Studies, University of Edinburgh, Easter Bush, Midlothian, United Kingdom; 2 MRC-University of Glasgow Centre for Virus Research, Glasgow, United Kingdom; 3 Institute of Technology, University of Tartu, Tartu, Estonia; 4 Centre for Immunity, Infection and Evolution, Ashworth Laboratories, University of Edinburgh, Edinburgh, United Kingdom; 5 Department of Entomology, University of Georgia, Athens, Georgia, United States of America; University of Minnesota, United States of America

## Abstract

Several components of the mosquito immune system including the RNA interference (RNAi), JAK/STAT, Toll and IMD pathways have previously been implicated in controlling arbovirus infections. In contrast, the role of the phenoloxidase (PO) cascade in mosquito antiviral immunity is unknown. Here we show that conditioned medium from the *Aedes albopictus*-derived U4.4 cell line contains a functional PO cascade, which is activated by the bacterium *Escherichia coli* and the arbovirus Semliki Forest virus (SFV) (*Togaviridae*; *Alphavirus*). Production of recombinant SFV expressing the PO cascade inhibitor Egf1.0 blocked PO activity in U4.4 cell- conditioned medium, which resulted in enhanced spread of SFV. Infection of adult female *Aedes aegypti* by feeding mosquitoes a bloodmeal containing Egf1.0-expressing SFV increased virus replication and mosquito mortality. Collectively, these results suggest the PO cascade of mosquitoes plays an important role in immune defence against arboviruses.

## Author Summary

Arboviruses are transmitted to vertebrates by arthropod vectors such as mosquitoes. Infection of mosquitoes with arboviruses activates immune defence responses including the RNA interference pathway. Another component of the insect immune system is the phenoloxidase (PO) cascade, which produces melanin that accumulates at wound sites and around invading microorganisms. Some pathogen-associated pattern recognition molecules are known to activate the PO cascade, which results in the proteolytic processing of inactive prophenoloxidase (PPO) to PO. PO then catalyses the formation of compounds that ultimately form melanin. Some of these products are also known to have anti-microbial properties but whether activation of the PO cascade provides any defence against arboviruses is unclear. Using the arbovirus, Semliki Forest virus, we show that this virus activates the PO cascade. By using recombinant Semliki Forest virus expressing an inhibitor of the PO cascade, we also demonstrate that this pathway inhibits virus spread in cell culture. Moreover, inhibition of this pathway leads to higher virus genome levels and higher mortality of infected mosquitoes. In conclusion, Semliki Forest virus activates the PO cascade which exhibits antiviral activity and can be added to the list of mosquito anti-viral defence mechanisms.

## Introduction

**Figure 1:**
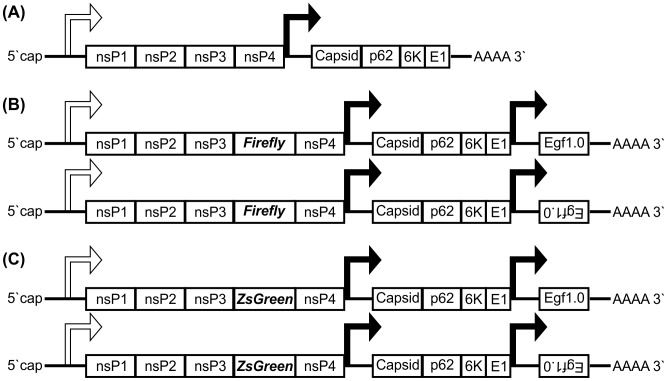
Viruses used in study. (**A**) SFV (prototype strain SFV4). (**B**) SFV(3H)-*FFLuc*-Egf1.0F and SFV(3H)-*FFLuc*-Egf1.0R, encoding Firefly luciferase (*FFLuc*) as part of the non-structural polyprotein (inserted between duplicated nsP2 cleavage sites at the nsP3/4 junction), and from a duplicated subgenomic promoter the melanisation inhibitor Egf1.0 in sense (F virus; top) or (as negative control) antisense orientation (R virus; bottom). (**C**) SFV(3F)-*ZsGreen*-Egf1.0F and SFV(3F)-*ZsGreen*-Egf1.0R, expressing ZsGreen inserted into the C-terminal region of nsP3, and from a duplicated subgenomic promoter the melanisation inhibitor Egf1.0 in sense (F virus; top) or (as negative control) antisense orientation (R virus; bottom).

The transmission of arboviruses by mosquitoes and other arthropod vectors has considerable adverse impacts on human and animal health. This group of pathogens consists primarily of viruses in the families *Flaviviridae*, *Togavirida*e *Bunyaviridae*, and *Reoviridae* [1]–[4]. Arboviruses replicate in both vertebrate and arthropod hosts. In mosquitoes, arboviruses must also spread from the midgut, which is the initial site of infection following a bloodmeal to the salivary glands for transmission to another vertebrate host. The genus *Alphavirus* (family *Togaviridae*) contains several mosquito-vectored arboviruses including models like Sindbis virus (SINV) and Semliki Forest virus (SFV) [5], [6] but also the re-emerging human pathogen chikungunya virus (CHIKV) [7]. The genetic structure and replication of alphaviruses, which replicate in the cytoplasm, have been analysed in detail [5], [6], [8], [9]. All members of the genus have positive-stranded RNA genomes that are approximately 11–12 kb in size, and have 5′ caps and 3′ poly(A) tails (genetic structure of SFV shown in Fig. 1A). All alphaviruses also encode two major polyproteins. The 5′ encoded non-structural polyprotein P1234 is proteolytically cleaved into replicase proteins nsP1–4 while the 3′ encoded structural polyprotein (translated from a subgenomic mRNA, which is transcribed under control of a subgenomic promoter) is proteolytically cleaved into the structural proteins that form the capsid and envelope of the virion. The glycosylated envelope proteins play key roles in entry into cells by mediating virus binding to host cell receptor(s) and subsequent fusion to endosomes (though other entry mechanisms may be possible) while the capsid protein encapsulates the viral genome [10]–[12]. Infection of mosquito cell cultures has also been useful to study arbovirus replication, thus allowing increasingly detailed studies of arbovirus/vector interactions [13], [14].

The innate immune system of mosquitoes plays an important role in the control of arbovirus infections, and SFV has proven to be a good models to study mosquito antiviral response mechanisms [14]. A key antiviral defence is RNAi (reviewed in [14]–[17]), which also influences arbovirus spread and transmission [18], [19]. In addition, differential regulation of mosquito immune signalling pathways and other host genes has been described following infection by dengue virus (DENV), West Nile virus (WNV) and SINV [20]–[24]. JAK/STAT and Toll signalling pathways both mediate antiviral activity against DENV [23], [24]. Interestingly, infection of *Anopheles gambiae* with the alphavirus o'nyong-nyong (ONNV) did not result in upregulation of the Toll and JAK/STAT pathways although other genes involved in immunity were upregulated with some displaying antiviral activities [25]. Innate immune signalling can also inhibit SFV replication in mosquito cells [26], while experiments in *Drosophila melanogaster* suggest that replication of SINV is inhibited by the IMD pathway [27].

Another conserved component of the insect immune system is the extracellular phenoloxidase (PO) cascade, which generates cytotoxic intermediates and the formation of melanin following wounding or infection [28]–[32]. Several factors have been shown to activate the PO cascade including pathogen-associated molecular pattern molecules like bacterial peptidoglycan. Other components of the cascade include multiple clip-domain serine proteases (cSPs) whose activation results in processing of the zymogen prophenoloxidase (PPO or proPO) to form active PO. PO then catalyses the conversion of mono- and di-phenolic substrates to quinones, which are converted to melanin.

A number of studies have shown that deposition of melanin provides defence against bacteria and multicellular parasites, while intermediates like 5,6-dihydroxyindole have been shown to be cytotoxic and act against pathogens [29], [33], [34]. Studies with the lepidopteran *Heliothis virescens* (tobacco budworm) indicate that haemolymph also contains factors with antiviral activity against *Helicoperva zea* single capsid nucleopolyhedrovirus (*Hz*SNPV) and other viruses including SINV, while bioassays with 5,6-dihydroxyindole show that it rapidly inactives *Autographa californica* multicapsid nucleopolyhedrosis virus (AcMNPV) [35]–[38]. Haemolymph melanisation in Lepidoptera also correlates with antiviral activity against *Microplitis demolitor* bracovirus (MdBV) [39], and *Lymantria dispar* multicapsid nucleopolyhedrovirus [40]. Whether arboviruses activate the PO cascade in mosquitoes and whether products of the PO cascade exhibit biologically relevant antiviral activity remains unclear, although interestingly RNAi knockdown of PPO I in the mosquito *Armigeres subalbatus* by a recombinant SINV expressing a dsRNA targeting PPO I resulted in reduced PO activity and higher SINV titres [41].

Previous studies show that *Aedes albopictus*-derived U4.4 cells have a functional antiviral RNAi response and immune signalling pathways [26], [42]. Here we show that conditioned medium from U4.4 cells contains inducible PO activity that is activated by exposure to bacteria and purified SFV particles. Expression of the PO cascade inhibitor Egf1.0 from MdBV [39], [43] by SFV decreased PO activity in U4.4 cell conditioned medium and enhanced the spread of virus through cell cultures. Infection of *Ae. aegypti* mosquitoes with SFV expressing Egf1.0 resulted in enhanced viral replication and mosquito mortality. Taken together, our results establish a role for the PO cascade in mosquito immune defence against an arbovirus.

## Results

### Immune challenge by bacteria and SFV increases PO activity in U4.4 cell-conditioned medium

The haemolymph of mosquitoes melanises in response to a variety of stimuli including wounding and infection [28]. Mosquitoes including *Ae. aegypti* encode multiple PPO genes, with some family members being inducibly expressed in response to microbial infection [44]–[47]. Haemocyte-like cell lines from *An. gambiae* also express multiple PPO genes [48], and recent studies identify cSP CLIPB9 as a candidate PAP [49].

**Figure 2:**
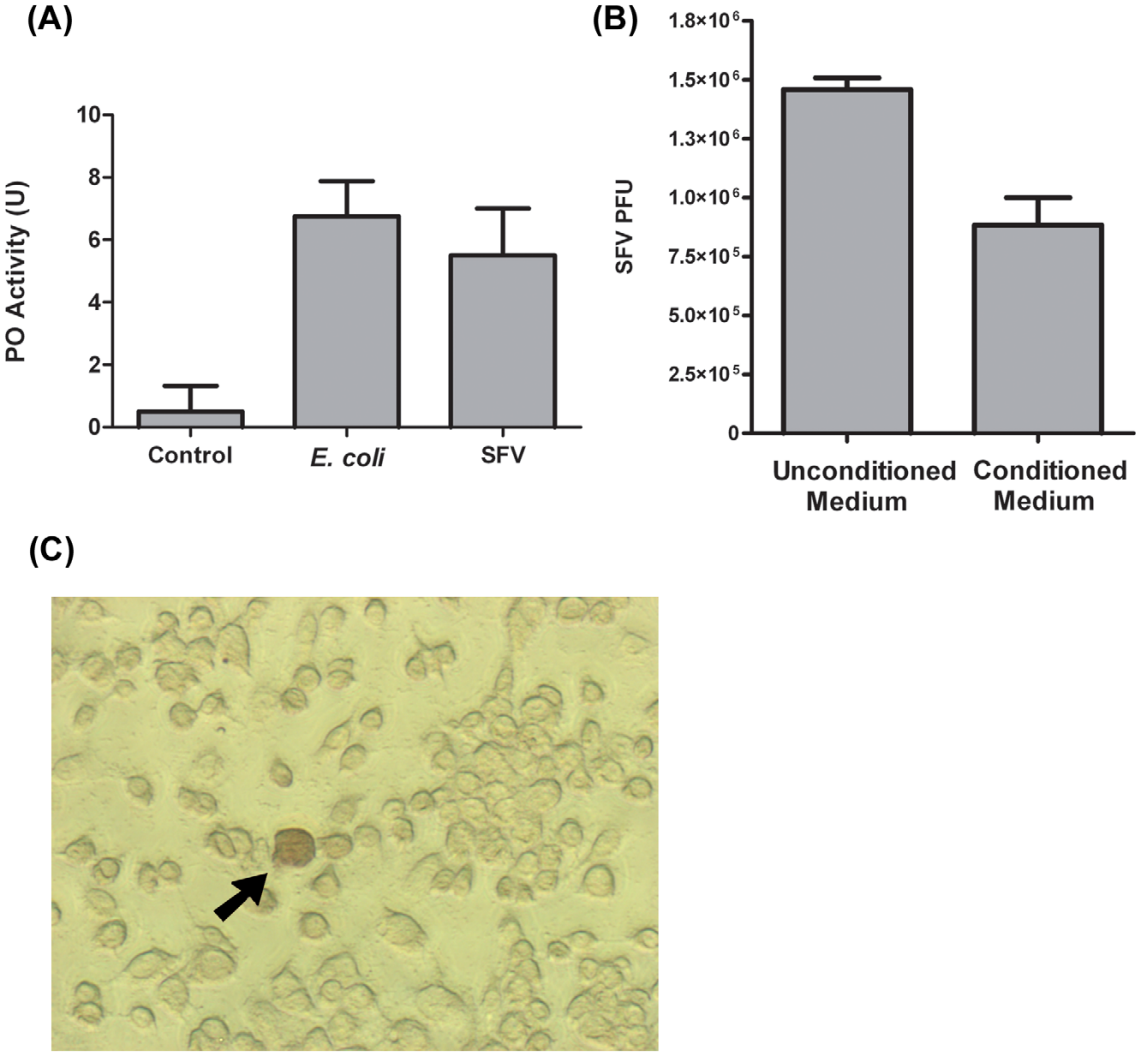
PO activity in U4.4 cell-conditioned medium. (**A**) PO activity in conditioned medium without immune challenge (Control) or after the addition of *E. coli* or purified SFV virions. One unit (U) of PO activity was defined as **Δ**A_490_ = 0.001 after 30 minutes incubation (see Materials and Methods). Each bar represents the mean from 10 reactions; error bars show standard deviation. This experiment was repeated three times with similar results. (**B**) SFV virion viability after a 1 h incubation at 28°C in unconditioned culture medium or medium conditioned by U4.4 cells for 48 h. Viability was then determined by titration of SFV on BHK-21 cells. PFU: plaque forming units. Each bar represents the mean from triplicate incubations; error bars show standard deviation. This experiment was repeated three times with similar results. (**C**) Staining for intracellular PO activity in U4.4 cells. Arrow indicates a U4.4 cell that melanised after fixation and incubation with the PO substrate dopamine. Note the larger size of this cell and its rounded morphology relative to surrounding cells that have not melanised.

Since the U4.4 cell line from *Ae. albopictus* is an important model for studying immune responses against arboviruses [26], [42], [50], we first asked whether conditioned medium from this cell line exhibited an increase in melanisation upon exposure to SFV or the bacterium *Escherichia coli* which is a well known elicitor of the PO cascade. Using a standard spectrophotometric assay for measuring melanisation activity (see Materials and Methods), our results indicated that PO activity significantly increased in U4.4 cell conditioned medium following exposure to each microbe (p = 0.003; *E. coli* versus control, p = 0.004; SFV versus control, p = 0.021; *E. coli* versus SFV, p = 1.00) (Fig. 2A). Our results also indicated that a 1 h incubation in conditioned medium significantly reduced SFV viability relative to virus incubated in unconditioned medium (p = 0.011) (Fig. 2B).

Because amphipathic molecules like detergents and alcohol activate insect PPOs [51], intracellular PO activity is commonly assayed for in PO producing cells like haemocytes by first fixing them in methanol and then incubating in a substrate like dopamine, which PO utilizes to produce melanin. This in turn causes the fixed cell to turn black or darken. In the case of *Ae. aegypti* and *An. gambiae*, prior studies establish that one class of haemocytes, oenocytoids, constitutively exhibit intracellular PO activity while a second class, granulocytes, inducibly exhibit intracellular PO activity following immune challenge with bacteria [52], [53]. To assess whether U4.4 cells exhibit intracellular PO activity, we fixed cells in glacial methanol and then incubated them in buffer plus dopamine. Our results showed no intracellular PO activity in the majority of cells but a small fraction of cells (0.2%) darkened in manner similar to mosquito haemocytes (Fig. 2C) [52], [53]. We also noted that these melanising cells display a rounded morphology and appear larger than other U4.4 cells that do not darken after fixation and incubation with substrate. We thus concluded from these assays that U4.4 cell-conditioned medium melanises following exposure to SFV or bacteria, and that a small proportion of U4.4 cells also melanise after fixation. We also concluded the increase in melanisation activity that occurs in conditioned medium correlates with a reduction in SFV viability.

### Expression of Egf1.0 by SFV inhibits PO activity in U4.4 cell-conditioned medium

**Figure 3:**
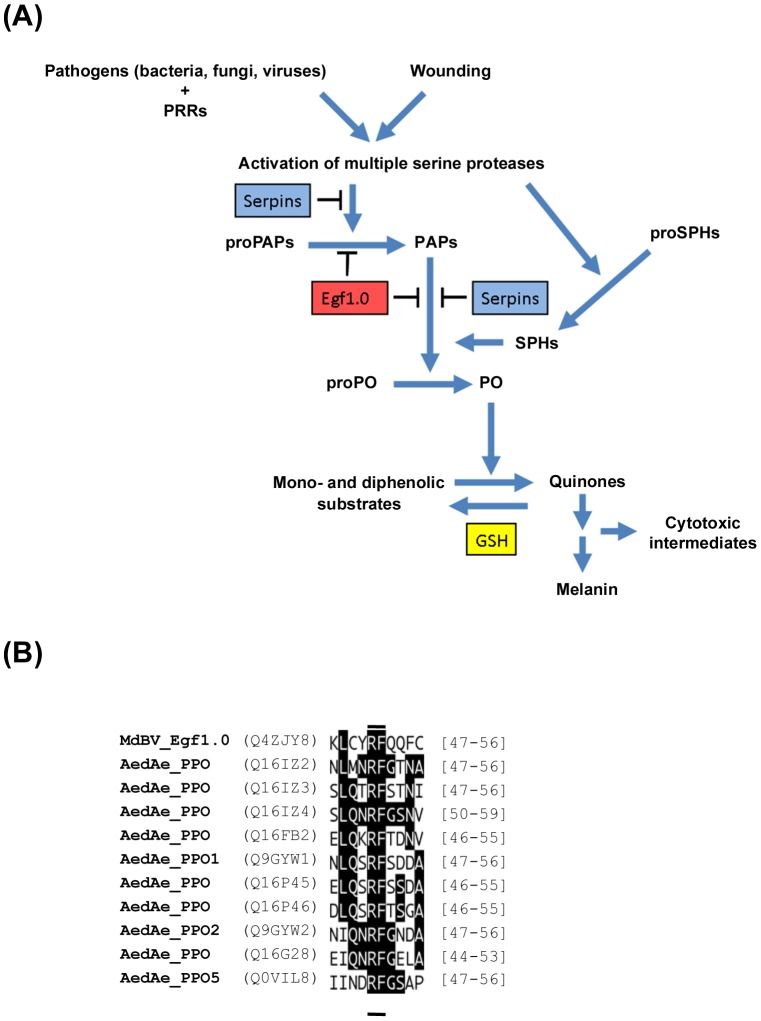
Activation and inhibition of the melanisation pathway. (**A**) Schematic showing the insect PO cascade and the known mode of action of Egf1.0. Infection by different pathogens and external wounding both trigger activation of the PO cascade. Proteins in the haemolymph known as humoral pattern recognition receptors (PRRs) bind to factors on the surface of different pathogens. This interaction as well as external wounding trigger the activation of multiple serine proteases. Some of these proteases have been identified in different insect species, whereas others remain unknown. Activation of these proteases leads to activation of prophenoloxidase activating proteases (proPAPs to PAPs). Some PAPs also require serine protease homologs (SPHs) for function, which are themselves activated by upstream serine proteases. PAPs cleave proPO (also called PPO) to PO, which oxidizes mono- and diphenolic substrates to quinones that undergo further reactions to form melanin. A number of these intermediate products are cytotoxic including some that have also been shown to inactivate viruses (see Text). Serine protease inhibitors called serpins have been identified from different species of insects that inhibit PAPs or other proteases in the pathway. In the absence of wounding or infection, the reducing agent glutathione (GSH) also exists in haemolymph at concentrations that can inhibit melanisation by recycling quinones to diphenols. The inhibitor Egf1.0 from MdBV inhibits both the processing of proPAPs and PAPs that have already been activated. (**B**) Alignment of the reactive site loop of Egf1.0 to the predicted cleavage sites for the PPOs encoded by *Ae. aegypti* (indicated as AedAePPO). Note the identical P1–P1′ residues R-F (underlined) of Egf1.0 and PPO family members. Black highlighting indicates identical residues. UniProt database identifiers in parentheses to the left of the alignment.

As previously noted, the PO cascade consists of multiple proteases that terminate with the zymogen PPO [28]–[32] (Fig. 3A). The number of proteolytic steps in the cascade has not been fully characterised in any insect including mosquitoes. However, it is known that infection, wounding, and other challenges trigger activation of upstream serine proteases, which result in processing of proPAPs (also referred to as pro-PPAEs or pro-PPAFs) between their clip and protease domains. Activated PAPs then process PPO by cleavage at a conserved arginine-phenylalanine (R-F) site in the N-terminal domain of the protein, which results in formation of PO (Fig. 3B). PO catalyses the hydroxylation of monophenols like tyrosine to *o*-diphenols and the oxidation of *o*-diphenols to quinones. Quinones thereafter undergo further enzymatic and non-enzymatic reactions that produce cytotoxic intermediates and ultimately melanin. Negative regulation of the PO cascade occurs through endogenous protease inhibitors like serpins, while reducing agents in haemolymph like glutathione (GSH) likely inhibit melanisation by reducing PO-generated quinones back to diphenols [54] (Fig. 3A). Several pathogenic organisms have also evolved strategies to suppress the PO cascade of hosts [28]. One of these is the virus MdBV, which produces the protein Egf1.0. Functional characterization of Egf1.0 showed that it blocks haemolymph melanisation in diverse insects including mosquitoes through two activities (Fig. 3A, B). First, it competitively inhibits activated PAPs because it contains an R-F reactive site that mimics the cleavage site for PPO [39]. Second, Egf1.0 contains another domain that prevents upstream proteases from processing pro-PAPs [43].

Given this background, we asked whether Egf1.0 could inhibit the increase in melanisation activity that occurs in U4.4 cell-conditioned medium following exposure to SFV or *E. coli*. To answer this question, we produced two sets of constructs. In the first, we cloned the *egf*1.0 gene from MdBV [39] in forward (expressing the Egf1.0 protein) and reverse (negative control not expressing Egf1.0) orientation into SFV under control of a second subgenomic promoter to produce SFV4(3H)-*FFLuc*-Egf1.0F and SFV4(3H)-*FFLuc*-Egf1.0R (Fig. 1B). These viruses also expressed Firefly luciferase (*FFLuc*), which served as an indicator for viral replication and spread through a U4.4 cell culture as previously shown for reporter gene-expressing SFV [50] (Fig. 1B). The second set of SFV constructs expressed Egf1.0 in forward or reverse orientation from a second subgenomic promoter plus ZsGreen fluorescent protein inserted into the C-terminal region of nsP3 to produce SFV4(3F)-*ZsGreen*-Egf1.0F and SFV4(3F)-*ZsGreen*-Egf1.0R, respectively (Fig. 1C).

**Figure 4:**
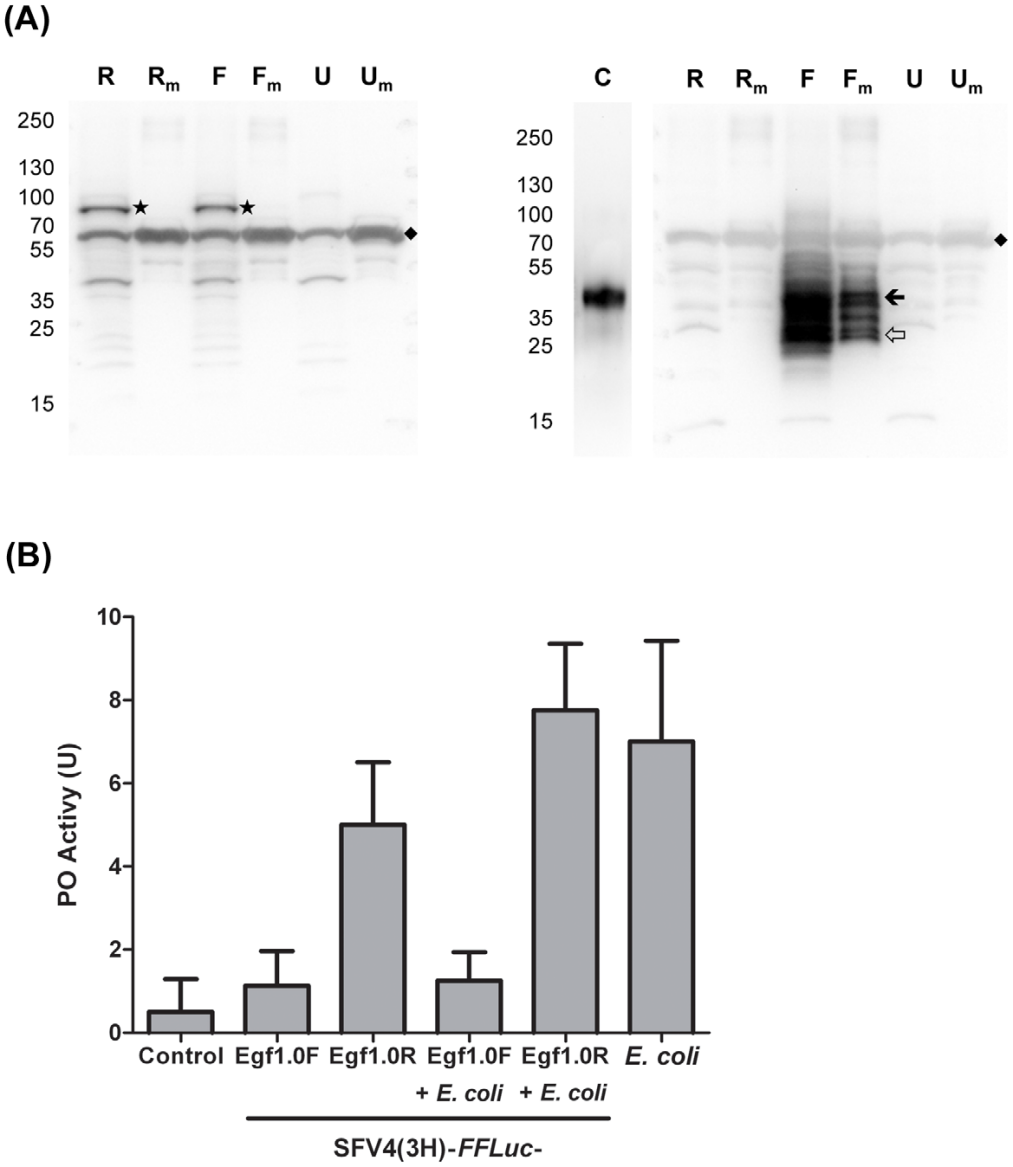
Recombinant SFV expresses Egf1.0 and inhibits PO activity in U4.4 cell-conditioned medium. (**A**) Immunoblots showing Egf1.0 expression and secretion from mosquito cells. U4.4 cells were infected with SFV4(3F)-*ZsGreen*-Egf1.0F or SFV4(3F)-*ZsGreen*-Egf1.0R at an MOI of 10 followed by preparation of cell lysate and medium samples at 48 h p.i. as indicated in the Materials and Methods. The left blot was probed with an anti-SFV nsP3 antibody with individual lanes labeled as follows: U4.4 cells infected with SFV4(3F)-*ZsGreen*-Egf1.0R (R = cell lysate, Rm = conditioned medium), U4.4 cells infected with SFV4(3F)-*ZsGreen*-Egf1.0F (F = cell lysate, Fm = conditioned medium), or uninfected cells (U = cell lysate, Um = conditioned medium). Black star identifies the nsP3-ZsGreen protein, only detected in lysates from SFV-infected cells. Black diamond indicates bovine serum albumin (non-specifically detected because of high abundance). The right blot shows the same samples probed with an anti-Egf1.0 antibody. A control lane (C) was added to this blot (purified, recombinant Egf1.0). Note that Egf1.0 is only detected in the control lane, and F and Fm lanes. Black arrow indicates uncut Egf1.0; open arrow identifies a band corresponding to the predicted C-terminal domain of Egf1.0 after PAP cleavage. Molecular mass markers indicated to the left. (**B**) PO activity in conditioned medium from uninfected U4.4 cells (Control), cells infected with SFV4(3H)-*FFLuc*-Egf1.0F (Egf1.0F), SFV4(3H)-*FFLuc*-Egf1.0R (Egf1.0R), cells infected with SFV4(3H)-*FFLuc*-Egf1.0F with *E. coli* added to the medium (Egf1.0F+*E. coli*), cells infected with SFV4(3H)-*FFLuc*-Egf1.0R with *E. coli* added to the medium (Egf1.0R+*E. coli*), or medium from uninfected cells with *E. coli* added (*E. coli*). PO activity was measured as outlined in Fig. 2A; 1 ml of conditioned medium was taken at 48 h p.i. from 2.6×10^5^ U4.4 cells infected at an MOI of 10, or uninfected (Control). Each bar represents the mean from 10 reactions; error bars show standard deviation. This experiment was repeated three times with similar results.

Next, the properties of SFV-expressed Egf1.0 were analysed. We infected U4.4 cells with SFV4(3F)-*ZsGreen*-Egf1.0F and SFV4(3F)-*ZsGreen*-Egf1.0R at a multiplicity of infection (MOI) of 10. Immunoblot analysis of cell lysates confirmed that each recombinant virus actively replicated as evidenced by detection of the nsP3-ZsGreen protein (Fig. 4A). Using an anti-Egf1.0 antibody, we also detected full-length Egf1.0 [39], [43] in the medium and lysates prepared from U4.4 cells infected with SFV4(3F)-*ZsGreen*-Egf1.0F but did not detect this protein in medium or lysates from uninfected cells or cells infected with SFV4(3F)-*ZsGreen*-Egf1.0R (Fig. 4A). Our Egf1.0 antibody also detected several other bands smaller than full-length Egf1.0 in samples infected with SFV4(3F)-*ZsGreen*-Egf1.0F including a 17.6 kDa protein that corresponded to the size of the C-terminal Egf1.0 fragment that prior studies showed is produced after cleavage by a PAP (Fig. 4A). Expression of Egf1.0 by SFV4(3H)-*FFLuc*-Egf1.0F and absence of Egf1.0 expression by SFV4(3H)-*FFLuc*-Egf1.0R were also verified by immunoblotting (not shown).

We then analyzed the functional properties of SFV-expressed Egf1.0 in conditioned medium from U4.4 cells. Melanisation assays at 48 h post-infection (p.i.) showed that conditioned medium from cells infected with SFV4(3H)-*FFLuc*-Egf1.0F exhibited very low PO activity, which was very similar and not significantly different to conditioned medium from uninfected (control) U4.4 cells (p = 1.0) (Fig. 4B). In contrast, medium from cells infected with SFV4(3H)-*FFLuc*-Egf1.0R exhibited PO activity levels that were significantly higher than medium from uninfected control cells (p = 0.025) (Fig. 4B). Conditioned medium of U4.4 cells infected with SFV4(3H)-*FFLuc*-Egf1.0F also contained significantly less (75%) PO activity than medium from cells infected with control virus SFV4(3H)-*FFLuc*-Egf1.0R (p<0.001) (Fig. 4B). The addition of *E. coli* to medium from SFV- infected cells had no effect on the PO activity (p = 0.251). As shown in Fig. 4B, the addition of *E. coli* to medium from SFV4(3H)-*FFLuc*-Egf1.0F-infected cells did not increase PO activity as would be expected if Egf1.0 was inhibiting PAP activity. Addition of *E. coli* to medium from SFV4(3H)-*FFLuc*-Egf1.0R-infected cells also did not elevate PO activity beyond the elevated level of activity that already existed. Taken together, these results showed that SFV4(3H)-*FFLuc*-Egf1.0F produced Egf1.0 in U4.4 cells, which is secreted into the medium. Given prior evidence that Egf1.0 specifically inhibits the PO cascade by disabling PAP function, these data also strongly suggested that U4.4 cell-conditioned medium contains a functional PO cascade, which is activated by SFV or gram-negative bacteria, and which is inhibited by SFV-produced Egf1.0.

### The inhibitor Egf1.0 enhances SFV spread through U4.4 cell culture

**Figure 5:**
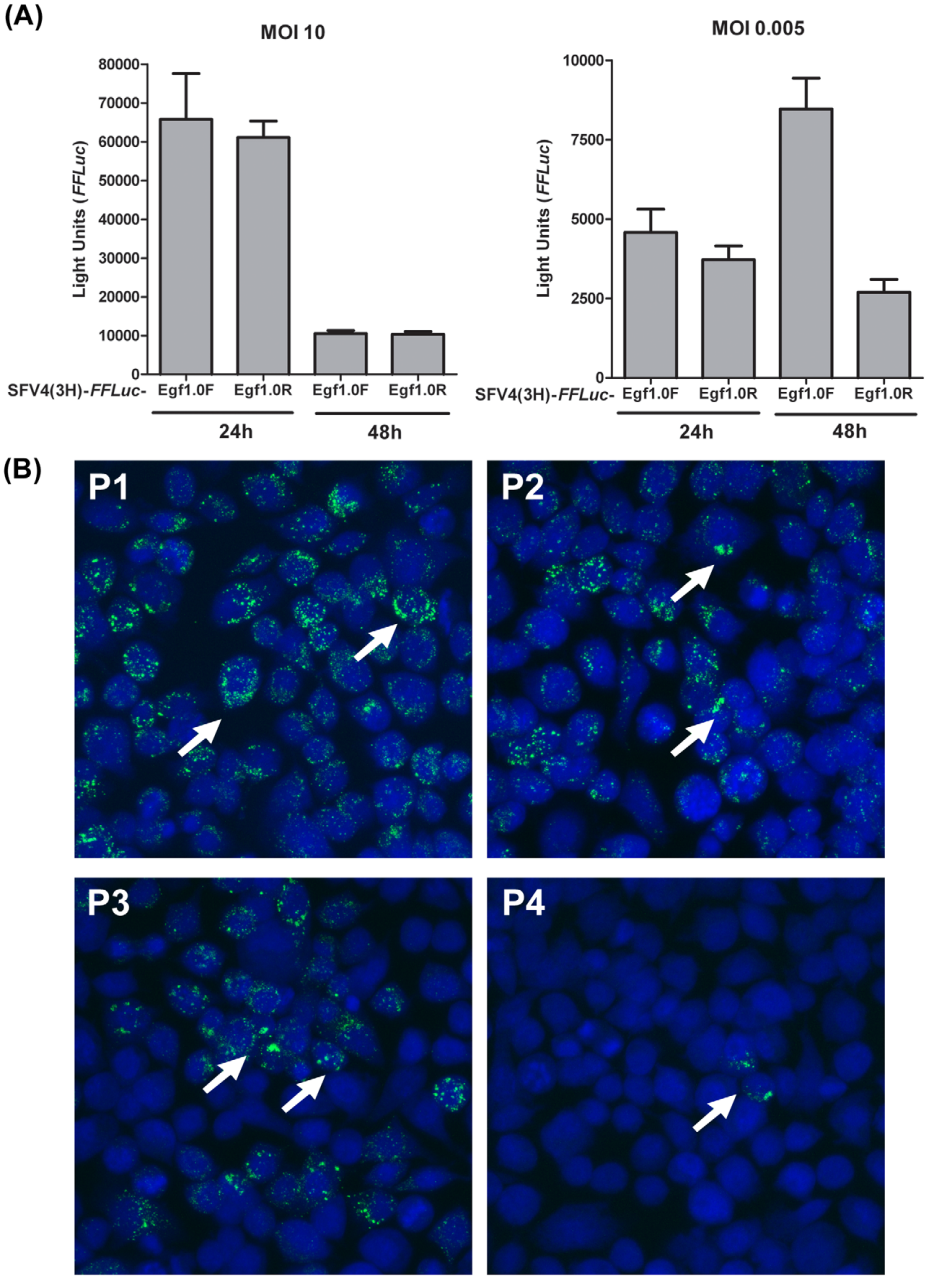
Inhibition of PO activity enhances the spread of SFV in U4.4 cells. (**A**) U4.4 cells were infected with SFV4(3H)-*FFLuc*-Egf1.0F (Egf1.0F) or SFV4(3H)-*FFLuc*-Egf1.0R (Egf1.0R) at an MOI of 10 (left graph) or 0.005 (right graph). *FFLuc* activity (expressed in lights units) was determined at 24 or 48 h p.i. Each bar represents the mean from triplicate cultures; error bars show standard deviation. This experiment was repeated three times with similar results. (**B**) Epifluorescent micrographs of U4.4 cells 48 h p.i. with SFV4(3F)-*ZsGreen*-Egf1.0F at an MOI of 10 (P1), SFV4(3F)-*ZsGreen*-Egf1.0F at an MOI of 0.005 (P3), SFV4(3F)-*ZsGreen*-Egf1.0R at an MOI of 10 (P2), or SFV4(3F)-*ZsGreen*-Egf1.0R at an MOI of 0.005 (P4). Arrows point to ZsGreen-positive replication foci in the cytoplasm of selected U4.4 cells. Cell nuclei are counterstained with marker TOPRO3. Note at low MOI infection the increased number of replication foci in cells infected with SFV4(3F)-*ZsGreen*-Egf1.0F (P3) relative to cells infected with SFV4(3F)-*ZsGreen*-Egf1.0R (P4). This experiment was repeated three times with similar results.

We next asked whether inhibition of PO activity by Egf1.0 could enhance virus spread during an infection. We first used our SFV4(3H)-*FFLuc*-Egf1.0F or SFV4(3H)-*FFLuc*-Egf1.0R constructs which allowed us to monitor viral replication and spread through a U4.4 cell culture by measuring *FFluc* activity at 24 h and 48 h p.i., similar to previously described experiments [50]. Infections were carried out at either a high multiplicity of infection (MOI 10), where most U4.4 cells were infected and little or no further spread of virus could occur, or a low MOI (0.005) where only a small fraction of cells were initially infected and SFV could thereafter disseminate through the medium to infect other cells. Overall GLM revealed differences in *FFLuc* activity as a function of MOI (10 or 0.005), construct (SFV4(3H)-*FFLuc*-Egf1.0F or SFV4(3H)-*FFLuc*-Egf1.0R) and sample time (24 h or 48 h p.i.) (Fig. 5 A&B, p = 0.012). As a result the data from the high and low MOI treatments were examined separately.

At an MOI of 10, cells infected with SFV4(3H)-*FFLuc*-Egf1.0F or SFV4(3H)-*FFLuc*-Egf1.0R exhibited similar levels of *FFluc* activity at 24 h or 48 h p.i. (p = 0.74) (Fig. 5A). This outcome was fully consistent with most cells being infected and containing actively replicating SFV, while also indicating that Egf1.0 had no effect on intracellular replication activity. As expected, rates of replication also dropped to low levels for both recombinant viruses at 48 h p.i. (p<0.001) as they each entered the persistent phase of infection [26] (Fig. 5A). In contrast, we observed a very different outcome when cells were infected at a low MOI where *FFluc* activity differed between cells infected with SFV4(3H)-*FFLuc*-Egf1.0F or SFV4(3H)-*FFLuc*-Egf1.0R. At 24 h p.i, there was no difference in *FFLuc* activity between cells infected with SFV4(3H)-*FFLuc*-Egf1.0F and SFV4(3H)-*FFLuc*-Egf1.0R (p = 0.37), but at 48 h p.i. SFV4(3H)-*FFLuc*-Egf1.0F showed significantly higher spread and replication rates than SFV4(3H)-*FFLuc*-Egf1.0R (p = 0.004) (Fig. 5A). We reasoned that this difference was also most likely linked to the time required for Egf1.0 to be expressed and secreted, and infectious SFV to be produced.

Repeating these experiments using SFV4(3F)-*ZsGreen*-Egf1.0F and SFV4(3F)-*ZsGreen*-Egf1.0R allowed us to visualize virus spread from one cell to another through the green fluorescing foci that form from ZsGreen presence in viral replication complexes (ZsGreen is inserted into the C-terminal region of nsP3; Fig. 1C). At a high MOI of 10, most U4.4 cells contained green foci at 48 h when infected with SFV4(3F)-*ZsGreen*-Egf1.0F or SFV4(3F)-*ZsGreen*-Egf1.0R (Fig. 5B). At a low MOI of 0.005, however, more cells exhibited green foci at 48 h p.i. when infected with SFV4(3F)-*ZsGreen*-Egf1.0F than SFV4(3F)-*ZsGreen*-Egf1.0R (Fig. 5B).

**Figure 6:**
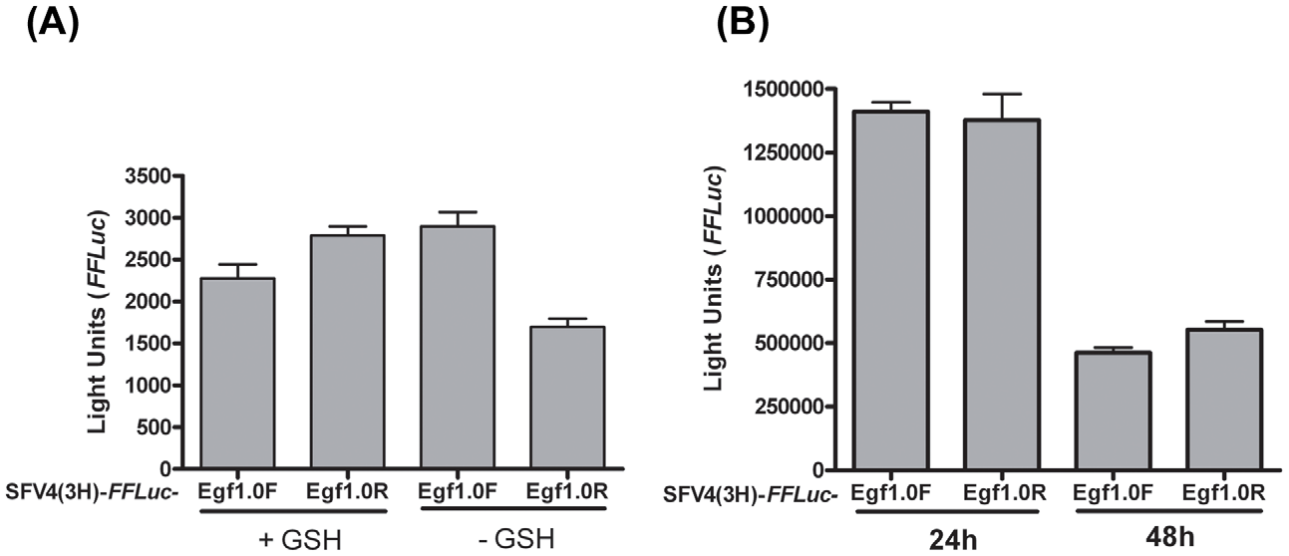
Spread of SFV in mosquito and vertebrate cells. (**A**) The addition of glutathione (GSH) to medium enhances the spread of SFV. U4.4 cells were infected with SFV4(3H)-*FFLuc*-Egf1.0F (Egf1.0F) or SFV4(3H)-*FFLuc*-Egf1.0R (Egf1.0R) at an MOI of 0.005 followed by determination of *FFLuc* activity at 48 h p.i. + GSH: 0.5 mM GSH; − GSH: negative control. Each bar represents the mean from triplicate cultures; error bars show standard deviation. This experiment was repeated three times with similar results. (**B**) Egf1.0 has no effect on SFV spread in BHK-21 cells. Cells were infected with SFV4(3H)-*FFLuc*-Egf1.0F (Egf1.0F) or SFV4(3H)-*FFLuc*-Egf1.0R (Egf1.0R) at an MOI of 0.005 followed by determination of *FFLuc* activity at 24 h and 48 h p.i. Each bar represents the mean from triplicate cultures; error bars show standard deviation. This experiment was repeated three times with similar results.

Overall, these data strongly suggested that activation of the PO cascade by SFV reduced virus spread, whereas Egf1.0 enhances virus spread by inhibiting the PO cascade. However, these results did not provide any insight into the identity of the effector molecules produced by the PO cascade that reduce SFV viability and spread. To assess whether the anti-SFV effects of PO were due to the formation of reactive intermediates or other products formed by PO, we infected U4.4 cells with a low MOI of SFV4(3H)-*FFLuc*-Egf1.0R (MOI 0.005) and added GSH (0.5 mM), which as noted above likely inhibits melanisation by reducing quinones (see Fig. 3A) [54]. Our results showed that GSH significantly increased the spread of SFV4-*FFLuc*-Egf1.0R relative to medium without added GSH (p<0.001). As expected though, the addition of GSH did not change the rate of spread of SFV4(3H)-*FFLuc*-Egf1.0F (p = 0.139) (Fig. 6A).

 Although vertebrates lack a PO cascade, we also tested whether expression of Egf1.0 conferred a replicative advantage to SFV in BHK-21 cells. There was no significant difference in the spread of SFV4(3H)-*FFLuc*-Egf1.0F and SFV4(3H)-*FFLuc*-Egf1.0R (p = 0.64) following low MOI infection (0.005), indicating that Egf1.0 had no effect on dissemination of SFV in this mammalian cell line (Fig. 6B).

### PO activity protects mosquitoes following SFV infection

**Figure 7:**
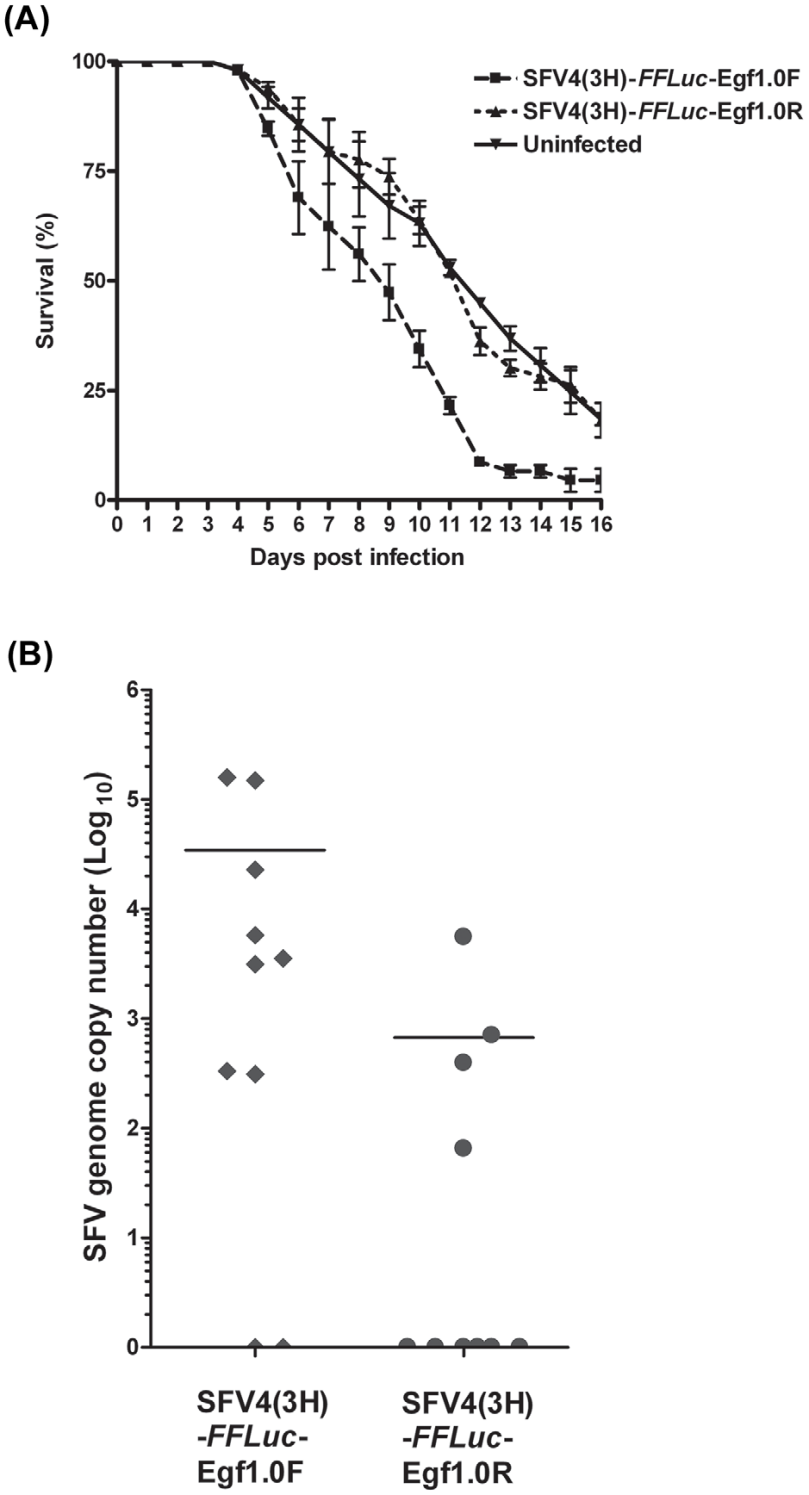
Expression of Egf1.0 increases mortality of *Ae. aegypti* and replication of SFV *in vivo*. (**A**) *Ae. aegypti* were fed blood containing SFV4(3H)-*FFLuc*-Egf1.0F or SFV4(3H)-*FFLuc*-Egf1.0R. Uninfected blood meals served as a control. Mosquito mortality was then monitored daily post-bloodmeal. Combined survival data from three independent experiments (cohorts of 22–25 infected mosquitoes per virus or control mosquitoes in each experiment) are shown. Error bars show standard deviation. (**B**) SFV genome copy number as determined by real time qPCR. Total RNA was extracted 3 days post-bloodmeal from mosquitoes infected with SFV4(3H)-*FFLuc*-Egf1.0F or SFV4(3H)-*FFLuc*-Egf1.0R. Viral genome RNA levels from 10 mosquitoes for each virus are shown. Values at 0 represent uninfected mosquitoes. Horizontal bar indicates average genome copy number from infected mosquitoes. This experiment was repeated three times with similar results.

Immunologically important antiviral pathways in mosquitoes such as RNAi have been previously implicated in promoting mosquito survival after arbovirus infection. Indeed, inhibition of the RNAi pathway through alphavirus-expressed RNAi inhibitors results in rapid death of virus-infected mosquitoes [55], [56]. To test whether the PO cascade provides an effective antiviral defence in mosquitoes, we extended our experiments to *Ae. aegypti*, a mosquito species that is generally relevant as an arbovirus vector, and which has also been shown to transmit SFV in the laboratory [57]–[59]. Prior studies also implicate *Ae. aegypti* alongside *Ae. africanus* as a natural vector of SFV [60]. *Ae. aegypti* were fed bloodmeals containing SFV4(3H)-*FFLuc*-Egf1.0F, SFV4(3H)-*FFLuc*-Egf1.0R, or no virus (mock-infection). We then monitored mosquito survival (cohorts of 22–25 mosquitoes) following infection in three independent experiments to determine survival rates (Fig. 7A). Since no significant differences were detected within treatments in the three experiments (p>0.05), the samples were pooled for further analysis. Overall, mosquito survival differed significantly among treatments (Kaplan Meier χ^2^ = 25.37; p<0.001). Post Hoc multiple comparison tests revealed no significant difference in survival rates between the mock-infected control and mosquitoes infected with SFV4(3H)-*FFLuc*-Egf1.0R (p = 0.98). In contrast, mosquitoes infected with SFV4(3H)-*FFLuc*-Egf1.0F exhibited higher mortality than mock-infected mosquitoes (p<0.001) or mosquitoes infected with SFV4(3H)-*FFLuc*-Egf1.0R (p<0.001). In conclusion, inhibition of the PO cascade decreased survival following infection of mosquitoes with SFV.

To assess whether the reduced survival of SFV4(3H)-*FFLuc*-Egf1.0F-infected mosquitoes was associated with enhanced viral replication, mosquitoes (cohorts of 10) were fed bloodmeals containing SFV4(3H)-*FFLuc*-Egf1.0F or SFV4(3H)-*FFLuc*-Egf1.0R. Total RNA was then extracted at 3 days post-bloodmeal followed by qPCR analysis to determine SFV genome copy number per individual. This time point was chosen because it just precedes quantifiable differences in mosquito survival, thus avoiding mortality-induced bias. Our results showed that viral genome copy numbers were higher in mosquitoes fed SFV4(3H)-*FFLuc*-Egf1.0F than in mosquitoes fed SFV4(3H)-*FFLuc*-Egf1.0R (Mann-Whitney test, p = 0.04) (Fig. 7B). Interestingly, infection rates were also higher when mosquitoes were infected with SFV4(3H)-*FFLuc*-Egf1.0F than SFV4(3H)-*FFLuc*-Egf1.0R (Fig. 7B). This suggests that Egf1.0-mediated inhibition of the PO cascade is also potentially important in establishment of an infection. Higher infection rates have been previously observed with alphaviruses expressing RNAi inhibitors or following silencing of antiviral RNAi genes during mosquito infection [55], [61].

## Discussion

Comparative genome analysis of different mosquito species reveals a noticeable expansion of PPO genes relative to other insects. For example, *An. gambiae* encodes nine PPOs while *Ae. aegypti* encodes ten. Expansion in the numbers of clip-domain serine proteases and serpins has also occurred [47]. The recent sequencing of the *Culex quinquefasciatus* genome reveals nine PPOs and thirty-two serpins, compared to originally twenty-three serpins in *Ae. aegypti* though recent studies and Vectorbase increase this number to twenty-six [45]–[47], [62]. Compared to other insects including *An. gambiae*, relatively little is known about regulation of the PO cascade in mosquitoes although recent studies in *Ae. aegypti* identify some of the processes involved [62]. Interestingly the cSP family also contains proteins with non-catalytic protease domain, so-called clip domain serine protease homologs (cSPHs), and both cSPs and cSPHs (as co-factors) are involved in melanisation reactions.

In *Ae. aegypti* and *An. gambiae*, cSPs and cSPHs are divided into five subfamilies called CLIP A, B, C, D and E [47]. Mainly CLIP B subfamily proteases are known (or suggested) to activate PPOs. Melanisation in *Ae. aegypti* was found to be regulated by protease inhibitor Serpins-1, -2 and -3 which regulate different cSPs [62]. In that study, two separate pathways leading to PPO cleavage were described; a first pathway linking Serpin-1 to (CLIP B subfamily members) Immune melanisation protease (IMP)-1 and IMP-2, and a second pathway linking Serpin-2 to Tissue melanisation protease (TMP) and IMP-1. Depletion of Serpin-2 leads to tissue melanisation and appears to be involved in activation of the Toll pathway, while depletion of Serpin-1 leads to immune responses against the parasite *Plasmodium gallinaceum* [62]. Other regulators of melanisation in *Ae. aegypti* such as CLSP2 (a modular protein consisting of C-type lectin and elastase-like domains) have been described [63]. Transcription of at least some PPO genes in *Ae. aegypti* is also regulated by the Toll pathway [44], thus linking different branches of the immune response.

Based on the antiviral activities of insect haemolymph [35], [36], we hypothesized that immune reactions induced by PO extend to arboviral infection of mosquitoes. Our experiments collectively indicate that U4.4 cell-conditioned medium contains a functional PO cascade. Our detection of a small proportion of U4.4 cells that melanise after fixation and incubation with dopamine further suggest these cells are likely source of the PO activity detected in conditioned medium. Notably, these cells morphologically resemble oenocytoids, which also comprise less than 1% of the circulating haemocyte population in mosquitoes like *Ae. aegypti* and *An. gambiae* [52], [53] as well as many other insects, yet are also the primary source of PO in plasma [51]. Ongoing analysis of the U4.4 cell transcriptome indicates that PPO orthologs are expressed although at this time it remains unclear whether expression is restricted to the large, rounded cells that stain after incubation with dopamine or is more global. Regardless of these uncertainties, our results strongly indicate that medium conditioned by U4.4 cells contains a functional PO cascade that is activated by exposure to SFV or *E. coli*, and is inhibited by Egf1.0. Prior studies in Lepidoptera show that MdBV also activates the PO cascade [39] while bacterial cell wall components like peptidoglycan are well known activators of the PO cascade in a diversity of insects [28]–[32]. We think it likely that activation of the PO cascade in U4.4 cell-conditioned medium by *E. coli* similarly involves binding of bacterial cell wall components by currently unknown humoral pattern recognition receptors. In contrast, it remains unclear what features of SFV induce a similar increase in PO activity. One possibility is that glycoproteins of the viral envelope function as pathogen-associated molecular patterns. The lectin pathway of vertebrate complement is known to be activated by pattern recognition receptors such as mannose–binding lectin that binds mannose-containing glycoproteins [64]. Several lectins have also been described as candidate pattern recognition receptors in insects [65].

While additional studies will be needed to identify how SFV is being recognised in U4.4 cell conditioned medium, our results collectively indicate that activation of the PO cascade and the associated increase in melanisation that occurs reduces the spread of SFV among the U4.4 cell population. Reduced survival of *Ae. aegypti* combined with enhanced virus replication when mosquitoes are infected by SFV expressing Egf1.0 also suggests the PO cascade is important in limiting arbovirus spread in mosquitoes. Interestingly, gene expression data obtained following ONNV infection of *An. gambiae* indirectly suggest that ONNV infection may have led to activation of melanisation pathways in the early stages of infection [25], which highlights the importance of this study.

On the other hand, the effects of PO cascade inhibition on mosquito survival are most apparent at later stages post-bloodmeal compared to experiments with alphaviruses expressing RNAi inhibitors [55], [56]. This suggests that inhibition of the PO cascade takes more time than disruption of RNAi or that this response is less powerful than RNAi in defence against arboviruses. However these experiments show that viral expression of an inhibitor is a viable strategy for inhibiting insect immune responses. Expression from the subgenomic promoter of recombinant SFV results in high levels of Egf1.0 and strong inhibitory activity, which may be difficult to achieve by just silencing a target gene through RNAi. Thus, an important goal for future studies will be to assess how inhibition of the PO cascade affects the spread of SFV in different tissues of mosquitoes as well as how the PO cascade may interact with other immune defence responses including the RNAi pathway.

Previous experiments where PPO I was silenced in *Ar. subalbatus* by expression of PPO I dsRNA using recombinant SINV showed increased titres of SINV [41]. Our results take this observation further by showing that activation of the PO cascade reduces SFV viability *in vitro* and that Egf1.0-mediated inhibition enhances virus replication and spread both *in vitro* and *in vivo*. However it is not entirely clear what products generated by the PO cascade are responsible for the antiviral activity against SFV we observe. Given the antiviral properties of 5,6-dihydroxyindole against AcMNPV [38], and the ability of GSH to inhibit anti-SFV activity in conditioned U4.4 cell culture medium suggests that the reactive intermediates generated by PO are antiviral. However, it is also possible the PO cascade might reduce arbovirus spread from the initial site of infection through the production of melanin and/or activation of other signaling pathways like Toll or IMD that also have roles in antiviral defence. To distinguish between these possibilities will require studies that directly assess the effects of 5,6-dihydroxyindole, melanin, or other compounds on the integrity of SFV virions [38]. Any damage to structural proteins could result in failure to bind receptors and/or enter cells. Questions also remain over the tissue specificity of PO activity. Our *in vitro* and *in vivo* data overall suggest products of the PO cascade may be antiviral because they reduce the viability of virions in the haemocoel. However other research describes melanisation reactions in the extracellular space between *An. gambiae* midgut cells following *Plasmodium berghei* infection [66]. Thus inhibition of PO activity by Egf1.0 could enhance SFV replication and spread in or around midgut tissues. Finally, our study does not directly address the question of whether wild-type SFV can potentially inhibit or evade the PO response. Given though that SFV spread is enhanced by expression of a powerful inhibitor like Egf1.0, we suspect the ability of wild-type SFV to inhibit or evade host-associated PO defence response is likely weak.

Alphaviruses are not inhibited by the Toll pathway in insects [26], [27], but links between the PO cascade and Toll signalling in *Ae. aegypti* could, as noted above, play a role in antiviral defence. Infection of *Ae. aegypti* with DENV-2 results in differential regulation of serpins although it is not possible yet to speculate whether these have a role in controlling PPO activation [24]. It does however suggest that protease-mediated antiviral defences extend to other arbovirus families. Intriguingly, it has been shown that infection of insects with strains of endosymbyotic *Wolbachia* bacteria, which can inhibit arbovirus infection by yet unknown mechanisms [67], may upregulate melanisation or genes involved in melanisation [68], [69]. Thus, our findings also may explain in part the antiviral properties mediated by *Wolbachia* infection. Future work will determine whether these findings also extend to viruses from other arbovirus families.

## Materials and Methods

### Ethics statement

Under UK Home Office legislation insects such as mosquitoes are not considered animals. No animals were used in the course of these experiments. Defibrinated sheep blood was obtained from TCS Biosciences (Buckingham, United Kingdom).

### Cells, viruses and infection

*Ae. albopictus*-derived U4.4 mosquito cells were grown at 28°C in L-15 medium with 10% fetal calf serum and 10% tryptose phosphate broth. BHK-21 cells were grown in Glasgow minimum essential medium (GMEM) with 10% newborn calf serum and 10% tryptose phosphate broth at 37°C in a 5% CO_2_ atmosphere. Amplification of SFV (strain SFV4) and recombinant clones derived from SFV4 in BHK-21 cells (grown as described above), together with titration of plaque forming units (PFU) in BHK-21 cells have been previously described [50]. SFV and derived clones were purified from supernatant as described and resuspended in TNE (Tris-NaCl-EDTA) buffer [70]. Viruses were diluted in PBSA (PBS with 0.75% bovine serum albumin) and added to U4.4 cells at room temperature for 1 h followed by washing twice to remove any unbound particles; cells were grown at 28°C following infection. Details of reporter viruses (Fig. 1) can be obtained from the authors. The pCMV-SFV4 backbone for production of SFV4 has been previously described [71]. A second subgenomic promoter was placed behind the SFV4 structural open reading frame for construction of viruses with duplicated subgenomic promoters [72]. This second subgenomic promoter is of the T37/17 type (consisting of a sequence 37 nucleotides upstream and 17 nucleotides downstream of the original transcription start-site of the SFV subgenomic mRNA). The ZsGreen marker was inserted into the C-terminal region of nsP3 via a *Xho*I site naturally occuring in the genomic sequence (leading to expression of nsP3 containing ZsGreen), while Firefly luciferase (*FFLuc*) was inserted between duplicated nsP2 cleavage sites at the nsP3/4 junction as a cleavable reporter, using strategies previously shown [73]. The full *egf1.0* coding sequence (including signal peptide) derived from MdBV was placed under control of the second subgenomic promoter in sense or antisense (as negative control) orientation.

### Detection of ZsGreen expression

Cells on glass slides were fixed in 10% formaldehyde (Fisher Chemicals) for 45 min and washed in PBS three times. Cells were treated with TO-PRO 3 (Invitrogen) (1∶1000) in dH2O for 10 min and washed with PBS three times. Slides were mounted using Vectashield mounting medium (Vector Laboratories). Cells and fluorescence were then visualised by confocal microscopy.

### Detection of Egf1.0 by immunoblotting

 At 48 h p.i., U4.4 cells infected with SFV (MOI of 10) or control uninfected cells were lysed in Laemmli buffer. Conditioned cell culture medium was concentrated on Millipore Centricon-Plus 70 Centrifugal Filter Units prior to addition of Laemmli buffer. Recombinant Egf1.0 produced as previously described [39] served as a positive control. Samples were run on a 4–20% Tris-Gycine PAGEr precast gels (Lonza), and blotted onto Immobilon-P PVDF membranes (Millipore). SFV infection was detected using a rabbit anti-nsP3 antibody (1∶20000), while Egf1.0 was detected using a rabbit anti-Egf1.0 antibody (1∶35000) [39], [74]. Primary antibodies were detected using a horseradish peroxidase (HRP)-conjugated goat-anti rabbit secondary antibody (Jackson ImmunoResearch) (1∶45000), followed by visualisation using the ECL Advance Western Blotting Kit (Amersham) and a GeneGnome bioimaging system (Syngene).

### Mosquito rearing and infection

*Aedes aegypti* (Liverpool red eye strain optimised for filarial growth) were kindly provided by R. M. Maizels and Y. Harcus (Institute of Immunology and Infection Research, University of Edinburgh). Mosquitoes were kept at 27°C, in 85% humidity and with a 16 h light: 8 h dark photoperiod. Larvae were fed on a standard yeast diet, while adults were fed on 10% fructose continuously. Female adults were 4 to 5 days old when allowed to feed on defibrinated sheep blood (TCS Biosciences) containing 5×10^7^ PFU of virus per ml of blood supplemented with 4 mM ATP. Mosquitoes were starved for 24 h before feeding and the bloodmeal (at 37°C) provided by a Hemotek membrane feeder (Discovery Workshops, Accrington, UK) for 2 h. Mosquitoes that fed were removed and maintained at standard conditions with fructose.

### Melanisation assays and determination of PO activity

 Conditioned cell culture medium from *Ae. albopictus*-derived U4.4 mosquito cells was harvested 48 h post-cell seeding (4×10^6^ cells in a 75 cm^2^ flask) and centrifuged at 2000 rpm for 5 min in order to eliminate residual cells. Approximately 5 µl of a pelleted *E. coli* JM109 culture (New England Biolabs) or 3.5×10^7^ PFU of SFV were added to 1 ml of cell culture medium and incubated for 10 min at room temperature. The mixture was then centrifuged at 3000 rpm for 10 min at 4°C in order to remove debris. Following this, PO activity assays were carried out in 96-well plates with 100 ul of 50 mM Sodium Phosphate buffer (pH 6.5) containing 2 mM dopamine added to 20 µl of cell culture medium [75]. PO activity was monitored by measuring absorbance at 490 nm using a plate reader (Dynatech MR5000) over a period of 30 min. It should be noted that this approach predominantly detects dopachrome and/or dopaminechrome rather than melanin itself. One unit of PO activity was defined as ΔA_490_ = 0.001 after 30 minutes, similar to previously described [39], [76], [77]. For each experimental condition, PO activities from 10 reactions were determined. Intracellular PO activity was assessed by first fixing U4.4 cells in glacial methanol. After rinsing in PBS, fixed cells were then incubated for 1 h in phosphate buffer plus 2 mM dopamine.

### Determination of luciferase activities

Following cell lysis in Passive Lysis Buffer (Promega), luciferase activities were determined by using a Dual Luciferase assay kit (Promega) on a GloMax 20/20 luminometer.

### Real time quantitative PCR analysis (qPCR)

SFV4 genome copy number was quantified as previously described [26]. Briefly, total RNA was isolated from single *Ae. aegypti* using Trizol (Invitrogen). RNA quality and quantity were assessed with a NanoDrop 1000 spectrophotometer (Fisher Scientific). A total of 0.5 µg of total RNA per mosquito was reverse transcribed using Superscript III kit (Invitrogen) and oligo-dT primer, and reactions were analysed in triplicate. The reaction mix contained 0.8 µM of each primer, FastStart SYBR Green Master x1 (Roche), and 2 µl of template. Tubes were heated to 94°C for 5 min, and then cycled through 94°C for 20 sec, 62°C for 20 sec, and 72°C for 20 sec for 40 cycles on a RotorGene 3000 instrument (Corbett Research). Sequences of the primers were as indicated: 5′ -GCAAGAGGCAAACGAACAGA-3′ (SFV-nsP3-for) and 5′ –GGGAAAAGATGAGCAAACCA-3′ (SFV-nsP3-rev). The number of SFV genome copies was calculated using a standard curve generated with the plasmid pSFV1.

### Statistical analysis

Data with 2 groups were analysed using either t-test or Mann Whitney tests, depending on the structure of the data. Data with more than 2 groups was analysed using General Linear Models (GLM). All GLMs were initially performed including all fixed effects and their interactions. Any post hoc tests were adjusted for multiple comparisons using the Bonferroni correction. Survival analysis was performed on cohorts of 22–25 mosquitoes. Differences between survivorship curves were tested using Kaplan-Meier estimator and the log-rank test. Where appropriate, multiple comparisons were performed and the Bonferroni correction was applied. All analyses were conducted using SAS v9.1.3 (SAS Institute Inc., Cary, NC, USA). Diagnostics were performed and plots of residuals were examined, confirming the goodness-of-fit of all models. Prior to analysis, it was specified that results with p<0.05 would be reported as exhibiting formal statistical significance.
